# Impact of Rhinovirus Infections in Children

**DOI:** 10.3390/v11060521

**Published:** 2019-06-05

**Authors:** Silvia Vandini, Carlotta Biagi, Maximilian Fischer, Marcello Lanari

**Affiliations:** 1 Pediatric Unit, Imola Hospital, 40026 Imola, Italy; s.vandini@ausl.imola.bo.it; 2 Pediatric Emergency Unit, Department of Medical and Surgical Sciences (DIMEC), St. Orsola-Malpighi Hospital, University of Bologna, 40138 Bologna, Italy

**Keywords:** Rhinovirus, bronchiolitis, asthma, wheezing, immune response

## Abstract

Rhinovirus (RV) is an RNA virus that causes more than 50% of upper respiratory tract infections in humans worldwide. Together with Respiratory Syncytial Virus, RV is one of the leading causes of viral bronchiolitis in infants and the most common virus associated with wheezing in children aged between one and two years. Because of its tremendous genetic diversity (>150 serotypes), the recurrence of RV infections each year is quite typical. Furthermore, because of its broad clinical spectrum, the clinical variability as well as the pathogenesis of RV infection are nowadays the subjects of an in-depth examination and have been the subject of several studies in the literature. In fact, the virus is responsible for direct cell cytotoxicity in only a small way, and it is now clearer than ever that it may act indirectly by triggering the release of active mediators by structural and inflammatory airway cells, causing the onset and/or the acute exacerbation of asthmatic events in predisposed children. In the present review, we aim to summarize the RV infection’s epidemiology, pathogenetic hypotheses, and available treatment options as well as its correlation with respiratory morbidity and mortality in the pediatric population.

## 1. Introduction

Rhinovirus (RV) has long been known to be the main etiologic agent of “common colds”, which are clinically characterized by an association of such signs and symptoms as rhinorrhea, nasal congestion, sore throat, cough, headache, and diffuse malaise. Overall, it is a relatively frequent but otherwise mild, self-limited syndrome. Therefore, its importance as possible causal factor of severe illness has often been neglected [1]. Nevertheless, in the last few decades, the impact of RV infection on the subsequent development of wheezing and asthma has been the subject of several studies. The following sections highlight the most recent discoveries concerning RV biochemical features as well as the related infection’s pathogenesis, the activation of host immunological response, the actual available therapeutic strategies, and RV’s correlation with respiratory morbidity in childhood.

## 2. Epidemiology

RV circulates worldwide. Infected healthy infants and adolescents are sometimes asymptomatic or may manifest mild signs and symptoms. Nevertheless, RV has been recognized as one of the most common respiratory viruses detected among patients affected by many infections, such as otitis media, croup, bronchiolitis, and pneumonia, and exacerbations of underlying chronic lung diseases [2,3,4,5,6]. In the world’s temperate climate zones, the peak incidence of RV infection usually occurs in the period from early fall to the middle to end of spring [7].

Most RV infections are acquired in the community. However, more and more nosocomial and/or health-associated outbreaks have recently been reported, and have affected both patients and sanitary staff members [8]. RV transmission generally occurs through direct exposure to, and inhalation of, respiratory droplets/micro-droplets, even though it can also take place via fomites (contaminated surfaces), including direct person-to-person contact, since RV has moderate resistance to alcohol hand rubs and to other common disinfectants [9]. The growing use of multiplex diagnostic platforms has allowed for us to have deeper insight into the causal relationship between respiratory diseases and RV, which has been commonly identified as “additional/co-pathogen” by recent epidemiologic data, especially in young children with acute Respiratory Tract Infection (RTI) [10]. A rising number of studies [11,12,13,14] report RV involvement to be the main or a significant etiologic agent in more than 50% of upper respiratory tract infections (URTIs), as well as to have a potential role as a trigger of asthmatic flares in both adults and children, highlighting the fact that this viral pathogen causes greater morbidity than previously recognized. RV is acknowledged to be the second most frequent cause of viral bronchiolitis, after Respiratory Syncytial virus (RSV), and it is probably deeply involved in the development of wheezing during the first years of life, as reported in several studies [15,16,17,18,19,20]. To date, approximately 20–40% of infants under one year of age that have been diagnosed with bronchiolitis seem to be infected or co-infected by RV, a rate that rises up to about 50% when considering hospitalized infants under three years of age [21].

A recent European study conducted on paediatric cases (persons under 14 years of age) of Community-Acquired Pneumonia (CAP), as confirmed by a Chest X-ray, reported the isolation of RV in 29% of the examined population, with 40% of them as a concurrent agent of two or more virus species [10]. Multiple viral infection was reported as the most prevalent etiology in toddlers, while clinical severity was not apparently increased in this age group. A nested study in the context of the broader and well-known Etiology of Pneumonia in the Community Study (EPIC) analyzed the 13 most commonly isolated respiratory viruses and noted a 24.4% isolation rate for one or more viruses from asymptomatic children [22]. At the same time, literature data show that the RV wheezing illness represents a strong predictor of subsequent wheezing/asthma, especially in two types of high-risk cohorts: early wheezers with an atopic background and/or pediatric patients hospitalized for early onset wheezing consequences [23,24,25,26]. Nevertheless, the real role of RV in otherwise healthy infants lacking an a priori increased risk of asthma is still unknown. One study, conducted by De Winter et al. [27] on a low-risk cohort (no family history of asthma, no prior hospitalization for infant respiratory/wheezing illness), suggested that RV-induced wheezing in early life might be a risk factor for subsequent development of wheezing/asthma in high-risk, but also in low-risk, populations of children. The results of these studies demonstrated that RV causes less structural damage in the airways than RSV, but it may determine bronchial hyper-reactiveness in predisposed patients; in both cases, the interactions among the viral load, pathogenicity, and the genetic features of the host (i.e., family history of allergy and atopy and environmental factors, including airway microbiome and exposure to tobacco smoke or pollution) influence the onset and severity of subsequent wheezing and asthma disease [23,24,25,26,27]. High-risk birth cohort studies report that RV-induced bronchiolitis and wheezing are a strong risk factor for school-aged asthmatic illness [17,25,28,29]. These results were confirmed by a multicenter cohort study [30] analyzing 921 infants under one year of age: three different profiles of severe bronchiolitis were identified. Bronchiolitis RSV-negative (mostly due to RV) was related to a history of breathing disorder and/or eczema during infancy as well as to a higher blood eosinophils count.

## 3. Microbiological and Immunological Aspects

From a microbiological point of view, RV is currently included in the *Picornaviridae* family. Before the molecular era, RV was considered to be different from enteroviruses, since, unlike them, it is inactivated by acid. The actual availability of modern molecular techniques contributed to clarifying the genetic similarity between RV and enteroviruses and also between the various RV species. For example, RV 87 and enterovirus D68 are very similar from a genetic point of view and both are acid-sensitive.

**Figure 1:**
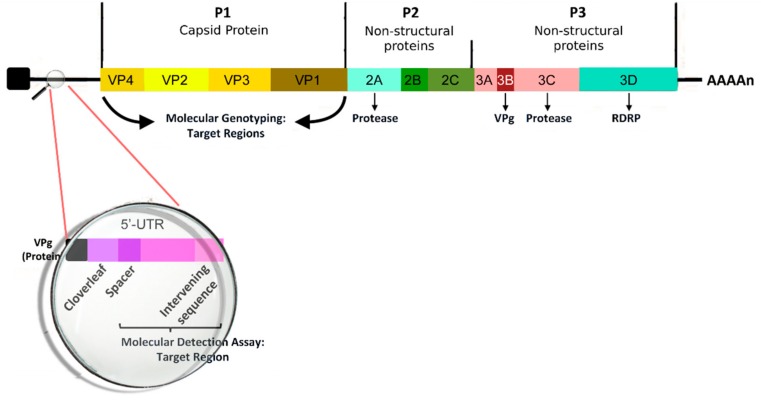
The genomic structure of Human Rhinovirus.

According to the latest ICTV release [31], we recognize three main RV species (RV-A, RV-B, and RV-C) under the genus Enterovirus, including also Enterovirus A e H and Enterovirus J. Furthermore, these specific species of RV account for more than 150 distinct serotypes [32]. RV is a non-enveloped, spherical virus with a mean diameter of about 30 nm. The viral capsid is composed of the four capsid proteins VP1, VP2, VP3, and VP4. The first three are present on the cell surface, while VP4 is found below the capsid. The icosahedral capsid contains a 7.2-kb positive-sense single-stranded RNA viral genome; several nonstructural proteins, such as 2A, 2B, 2C, 3A, 3B, 3C, and 3D, have been identified [33]. The genomic structure of RV is reported in Figure 1. The transmission of viral particles between humans is caused by direct contact or through the fomites [34]. RV survives on hands and surfaces for several hours, so human-to-human transmission is relatively frequent, particularly in the presence of high viral loads [35]. The airway epithelium is the primary site infected by RV; most RV-A and -B serotypes use Intercellular Adhesion Molecule (ICAM)-1 to enter inside the cell or may alternatively bind Low Density Lipoprotein Receptor (LDL-R), whereas RV-C generally acts by infecting the cells through a different receptor molecule section. A recent study [36] found no significant differences in the cytokine levels (IFN-γ, IL-4, IL-10, TGF-β) in a nasal sample during RV-A, B, or C infections. The mechanisms of response of the airway to RV infection have been extensively investigated because of their crucial rule in the complex and multifactorial process of development of asthma and recurrent wheezing at later ages. Unlike other respiratory viruses (i.e., RSV and Influenza Virus), RV alone does not cause direct airway epithelial cell destruction and does not have a definite cytopathic effect [37]. Instead, RV compromises the epithelial barrier’s function by dissociation of the zonula occludens-1 of the cells from the tight junction complex through the release of reactive oxygen species during viral replication [38]. In addition, the immune and adaptive immune response contributes to the pathogenesis of the infection through the activation of MDA1 and RIG1 genes and the synthesis and early secretion of IFNs and other pro-inflammatory cytokines (RANTES, IP-10, IL-6, IL-8, and ENA-78 [39]). Type I IFNs modulate the infection process through several infection control mechanisms [40], including the blocking of viral entry into cells, control of viral transcription, cleavage of RNA, inhibition of translation, and induction of apoptosis [41]. Moreover, IFNs indirectly mediate the production of cytokines and chemokines and the subsequent recruitment of natural killer cells and CD4 and CD8 T cells [42], the upregulation of the expression of major HLA I on cells, and upregulation of antigen-presenting cell mediators. Furthermore, RV infection also determines serotype-specific IgG neutralizing serum antibodies and IgA secretory antibodies in the airways, which are usually detectable one or two weeks after inoculation and maintain traceable levels for at least one year [39]. This humoral response seems to be serotype-specific, with only a minor fraction of antibody cross-reacting; for this reason, vaccine development is still very difficult. As reported in the literature, infected subjects with asthma show an increased number of lymphocytes and neutrophils in respiratory secretions and bronchial biopsy specimens whereas control subjects (without asthma) more often exhibit more lymphopenia associated with T cell infiltration of the airway’s epithelium and submucosa [43]. The findings of previous studies led some authors to hypothesize that immune responses against RV in the asthmatic patient may result in an inappropriate (delayed and/or deficient) process, with subsequent increased severity of post-infectious wheezing [44].

Several studies confirmed the role of interferons in susceptibility to asthma exacerbations in pediatric populations. A study by Miller et al. [45] analysed the role of upper viral infections in asthma exacerbations, demonstrating that RV was related to asthma exacerbation and was mainly mediated by an increased type III IFN response.

These results are similar to those reported in another study [46] that demonstrated that an increased susceptibility to severe respiratory viral infection during the first years of life is partly related to the development of dysfunctionality of key mechanisms that mediate innate immune defense, which manifests primarily as markedly consistent higher type III IFN responses. Another very recent study (Khoo et al., 2019 [47]) identified a new immunological phenotype related to different up- and downregulation of cytokines and different IFN types, with different susceptibility to asthma exacerbations.

A recent study conducted by Turi et al. [48] identified two different clusters of immune response to RSV and RV. One cluster, in common for both viruses, was characterized by increased type-2 and type-17 immune mediator (IL-4, IL-5, IL-13, IL-17, IL-20, IL-23, IL-33, and TSLP, a key regulator of asthma development) release, and it was linked to a higher risk of recurrent wheezing. In contrast, epithelial growth factor (EGF) appeared to be inversely related to the risk of subsequent development of respiratory morbidity, since it plays a key role in the tissue-repairing process. Moreover, advanced molecular diagnosis techniques are available to confirm these mechanisms, and they have been used to demonstrate that RV-A and RV-C are more often related to the onset of recurrent wheezing [49]. Another recent study, produced by Fedele et al., confirmed that a Th2-mediated immune response is more frequent after RV infection than after RSV infection, and this may explain the higher incidence of subsequent wheezing and asthma development after RV bronchiolitis [50].

Correlations between allergic sensitization and RV-induced wheezing are receiving more attention, and aim to clarify how a pre-existing risk for allergy and atopic disease affects the human immune response to RV infection and, at the same time, conversely how RV infection modifies the airway structure and organisms’ inflammatory state, increasing the risk of bronchial hyper-reactivity and wheezing [17]. For example, some studies have demonstrated that both RV infection and direct exposure to allergens cause, in the airways, epithelial cell production of IL-25 and IL-33, which are mediators involved in the type 2 airway inflammation and remodeling process [51,52]. Furthermore, the IL-33 polymorphism is significantly associated with the risk of the induction of intermediate and late-onset wheezing and allergic sensitization. However, it is still controversial whether genetic factors affect the immune response and subsequent airway reactivity and inflammation. The first barrier against RV infection is the airway epithelium, which is relatively resistant to infection when undamaged. In contrast, a disrupted airway epithelium may favor viral entrance in deeper cell layers, where RV has been demonstrated to replicate more actively. A damaged airway epithelium can also allow for the absorption of a higher amount of aeroallergens [53]. Moreover, RV can contribute to the airway remodeling process’s activation and maintenance by upregulating molecules, such as Vascular Endothelial Growth Factor (VEGF), TGF-β, and any type of chemoattractant for airway smooth muscle cells. This process may be more intense in young infants [54,55,56]. As recently described in a paper by Shariff et al. [54], recurrent RV infections are a strong stimulus for airway remodeling through an increase in smooth muscle cell mass recruitment next to the epithelial cells mediated by chemotactic molecules, such as CCL5, CXCL8, and CXCL10, which are secreted during RV infections. The mechanism of airway remodeling that is induced by RV was also described in a study by Leigh et al. [55]: RV infection determines the upregulation of such molecules as amphiregulin, activin A, and VEGF, which are notoriously involved in the remodeling of bronchial epithelial cells. Moreover, a recent study run on animal neonatal models by Hong et al. demonstrated that RV infection acquired in early life stages in mice induced an IL-13- and IL-25-mediated Th2 immune response with parallel suppression of IFN-γ, IL-12, and tumor necrosis factor (TNF)-α [56]. These modifications were shown to play a crucial role in asthma development, with detrimental changes in airway homeostasis, consisting of innate lymphoid cell expansion, mucous hypersecretion, and airway responsiveness; these findings were detected especially when RV infection occurred in the first months of life. Indeed, early infections occurring during the neonatal period may trigger a Th2 immune response, with a negative impact on lung development, thereby exposing the patient to a significant risk of chronic respiratory disease [57]. Moreover, the literature reports that the developing immune system is not able to activate an efficient “adult-type” immune response. This mechanism is due to the need, during the first months of life, to maintain the preservation of the delicate balance between the onset of potentially damaging, pro-inflammatory Th1 responses and the less damaging Th2- and Treg-mediated responses. As recently discussed by Kollman et al. [58], the immunologic function in the neonatal period is the result of a complex interaction among autonomous cell immunity, nutrition immunity, antigen-specific immunity, and leukocyte immunity.

Moreover, a recent birth cohort study [59] reported that the risk of severe lower RTI and subsequent development of wheezing during early infancy is directly related to a low production of type I and III IFN by cord blood mononuclear cells in response to viral infections.

Th1 cells produce interferon-gamma, IL-2, and TNF-beta, which activate macrophages and are responsible for cell-mediated immunity and phagocyte-dependent protective responses. In contrast, Th2 cells produce IL-4, IL-5, IL-10, and IL-13, which are responsible for triggering strong antibody production, eosinophil activation, and the inhibition of several macrophage functions, thus providing phagocyte-independent protective responses. Studies conducted on animal models [60,61] show that IL-13 is strongly induced during RV infections in neonatal mice, but not in adult mice. Moreover, in the Th2 immune response an increased production of IL-4, IL-33, and IL-25 [53,57] was observed. Increased Th2 responses are clearly involved in the onset of asthma and other atopic conditions because IL-4 and IL-13 are required for IgE synthesis and IL-5 is necessary for the recruitment of eosinophils. Another very recent study [62], enrolling 1016 infants <12 months in 17 U.S. centers, confirms that high levels of cytokines inducing a Th2 immune response (IL-4, IL-5, IL-13, and Thymic Stromal Linphopoyetin) are significantly related to the onset of asthma in infants up to four years of age. The main mechanisms that may explain the inflammatory response’s intensification during RV infection could be the impairment of the primary airway barrier’s function through a physical injury [63]. Furthermore, a prospective study, run on a cohort of nearly 5000 infants in Argentina, demonstrated that IL-13 may play an important role in the incidence of life-threatening RV infection, while levels of IFN-γ, viral load, and RV group were not significantly associated with the severity of the disease [64]. Some studies used the human challenge model to investigate the mechanism of viral infection, the response to RV infection in asthmatic hosts, and the worsening of virus-induced asthma activity [65] with the aim of developing new therapeutic strategies to control virus-induced inflammation and prevent the exacerbation of already-characterized asthmatic and wheezing episodes. RV-A and RV-C are more often related to wheezing in asthma exacerbations than RV-B, considering young subjects; RV-C, on the other hand, was associated with a more severe exacerbation and a higher hospitalization rate [66,67], probably because of a faster viral replication rate process and/or stronger triggered cellular responses. To understand the peculiar pathogenic potential of RV-C serotypes, it is important to consider that CDHR3 (Cadherin-Related Family Member 3) has recently been recognized as a unique receptor for RV-C [29]. This gene was recently discovered as a susceptibility gene for asthma in early childhood. The product of its expression may act as a possible receptor for RV-C, explaining why, in genetically predisposed infants, RV bronchiolitis may trigger asthma and wheezing whereas bronchiolitis caused by other viruses may not. In infants younger than six months, the receptor CDHR3 is more highly expressed than in older infants. Furthermore, RV-C infects epithelial cells of the airways and triggers a consistent production of cytokines, such as IL-25 and IL-33, which activates a strong Th2 immune response.

Another locus geni (17q21) was recently discovered and linked to the development of wheezing as well as to the predisposition of asthmatic disease during the first months of life. Wheezing episodes during early-age stages seem indeed to be a stronger risk factor for asthma onset in children if 17q21 risk variants are present and this association is stronger for RV as compared to RSV infection [29,68]. Moreover, Pech et al. reported that RV infection may cause differential DNA methylation and subsequently differential mRNA expression in the epithelial cells of the affected airways. The same kind of DNA methylation modifications have been observed in many genes involved in anti-viral immune responses and in the pathogenesis of asthma [69].

A recent study [70] identifies six distinct patterns of cytokine production in response to RV infection, with major differences among patients that developed asthma, allergic sensitization, and lower RTIs during childhood. The risk of severe asthma is related to immune response, with the lowest interferon induction and the highest proinflammatory cytokine secretion. These results confirm the hypothesis that early-life sensitization against RV may lead to an increased risk of early onset asthma and recurrent wheezing. Therefore, the identification of these immunological profiles might be useful to understand in more detail the pathogenetic disease mechanisms, and subsequently to develop personalized approaches to therapy. A very recent study [71] identifies six clusters of nasal microbiota and hypothesizes about the influence of nasal microbiota during RV infection on virus load, host innate immune response, and clinical course. Some authors observed that RV did not play a major role in modifying the upper respiratory tract microbiota, although a direct correlation between rhinorrhea’s severity and increased alpha-diversity after infection is described. This correlation could be explained by a variation in the microbiota caused by increased rhinorrea. Moreover, some studies have demonstrated that RV infection is associated with the detection of concomitant pathogenic bacteria in the patient’s airways [72,73]. In particular, RV-A and RV-C infection may be characterized by a higher isolation in nasopharyngeal aspirates of *Haemophilus Influenzae* and *Moraexella Catharralis*, which are very common etiologic causes of acute respiratory infections [74]. These data confirm the metabolomic analysis comparing the nasopharingeal aspirate of infants during RV or RSV infection [75]. While, for the first case studies, the literature describes a relative abundance of *H. Influenzae* in the nasal aspirate, in the second situation, on the other hand, a higher presence of *S. Pneumoniae* was most frequently found.

These results offer novel findings and additional evidence for an in-depth analysis of the complex interaction between the microbial (virus and bacteria) features and the host’s immunologic system in the pathobiology of bronchiolitis, a notoriously heterogeneous clinical disease.

## 4. Antiviral Agents

To date, no antiviral treatment for RV infection has been approved. The large number of RV serotypes and their considerable genetic diversity represented for years a major obstacle to the development of antiviral agents. Moreover, the RNA polymerase of this virus is reported to be error-prone, which leads to frequent natural mutations and an increased risk of drug resistance. Finally, to be effective, antiviral agents need to be administered during the early stages of respiratory infection and, in this sense, the lack of an accurate rapid diagnostic test represents a significant obstacle in RV treatment [76]. Additionally, the high cost of developing drugs has limited the interest of pharmaceutical companies to work in this area. However, some antivirals have exhibited inhibitory activity against RV.

Capsid binders are one of the main groups of RV antiviral agents. These drugs work through insertion into the VP1 hydrophobic pocket underneath the floor of the canyon, a depression of the viral capsid surface involved in cell receptor binding. This process prevents the capsid conformation changes that are necessary for RV to access the host cell. Pirodavir was the first capsid binder demonstrated to be capable of preventing RV infection in a human challenge model. However, this drug was demonstrated to be active only if administered within 10 minutes after RV challenge, while no antiviral effects was observed if administered 24 h after RV challenge [77]. Another capsid binding agent is Pleconaril, which is the first antiviral against RV tested in clinical trials. In two parallel prospective, double-blinded, placebo-controlled studies [78], the administration of this drug within the first 24 h after illness onset was able to consistently reduce the duration of symptoms in comparison with the placebo group. Nevertheless, the clinical benefits showed a strong correlation with the infecting virus’s susceptibility to the drug [79]. One more capsid binder, Vapendavir, has been recently assessed in Phase II clinical trials, but its efficacy is still unclear [80]. The new discovery of RV-C type viruses, which have been proven to be lacking the accessible hydrophobic pocket, partially explains the limited efficacy of these drugs in intervention trials.

Another potential antiviral drug group against RV is protease inhibitors. These molecules act by preventing the cleavage of viral proteins, a crucial process required for an effective replication of RV into the infected host cell. One example is Rupintrivir, an inhibitor of the human RV 3C protease. This drug showed moderate in vitro antiviral activity against a range of different RV serotypes [81], including human RV C strains [82]. However, further in vivo trials are still required to test the actual efficacy of this and the other protease-inhibitors agents against RV.

A different strategy in preventing RV infection is the restriction of an RV bond to one of the three main host cell surface receptors (LDL-R, ICAM-1, or CDHR3). To date, only monoclonal antibodies specific for ICAM-1 have been tested. Even though these molecules have been demonstrated to have inhibiting potential towards RV replication in vitro, they showed limited in vivo efficacy in clinical trials in addition to a high cost [83]. Further and more detailed studies analyzing RV’s infection and replication cycle are needed in the coming years to find new effective treatments against this very common but potentially dangerous micro-organism.

## 5. Vaccines

For decades, the development of vaccines against RV was considered unrealizable. Theoretically, an efficient and highly specific neutralizing humoral response against RV infection should confer solid protection. Nevertheless, because of the high and rising number of newly recognized viral strains, little cross-reactivity can be elicited by neutralizing antibodies [84].

In the last few decades, two main strategies have been implemented to overcome the high antigenic diversity of RV types to induce a valid protective immunity against different strains [85].

The first strategy is the development of a polyvalent vaccine comprising multiple RV serotypes. A previous attempt with a formalin-inactivated decavalent whole virus vaccine showed a significant increase in neutralizing antibody levels, active against up to 40% of known serotypes [86]. As a huge number of distinct RV strains circulate simultaneously [87,88], a consistent number of virus antigenic molecules of various types should be contained in a polyvalent vaccine. Recently, a 50-valent inactivated RV vaccine, assembled with an alhydrogel adjuvant, was demonstrated to be immunogenic against approximately one-third of the circulating RV types in rhesus macaques, inducing broadly neutralizing responses [89]. Of note, a limitation of the study conducted by Lee and colleagues is that the authors did not include RV-C antigens, which represents a crucial problem for pediatric populations. Those new multivalent RV vaccine approaches now need to be tested in humans.

A second strategy to induce protective immunity against different RV serotypes is to develop subunit vaccines that consist of small but conserved regions of the RV molecular structure and connect it with an adjuvant able to enhance the T-cell response. The aim of this process is to increase the serological spectrum of RV coverage while limiting the number of necessary antigens. The VP1 and VP4 + VP2 (VP0) capsid regions are the most conserved viral capsid structures and provide promising vaccine targets [84]. In a mouse model, a recombinant RV16 VP0 vaccine in conjunction with a combination of incomplete Freund’s (IFA) and CpG adjuvants elicited a strong cross-reactive Th1 response within the strain [89].

Overall, the recent findings in molecular virology, our understanding of RV’s structure, and the identification of type-specific differences have led to a renewed interest in the development of vaccines against RV. The identification of highly conserved RV regions and the design of an adjuvant polyvalent RV vaccine increase our hopes for future anti-RV vaccine development and efficient “weapons” against the variety of RV-associated diseases, including the increased risk for recurrent wheezing and subsequent childhood asthma. In addition, the use of biologic response modifiers to enhance the host’s innate immune responses to RV has been recently investigated. A randomized placebo-controlled trial was conducted to test the hypothesis that the administration of inhaled IFN-β might attenuate asthma exacerbations caused by RV and other respiratory viruses in patients with asthma after the onset of common cold symptoms [90]. Although the trial did not show a significant beneficial effect on asthmatic symptoms in the whole enrolled population, IFN-β was demonstrated to have a positive effect on morning lung function (peak expiratory flow), and it was able to boost innate immunity response to respiratory viruses, both locally in the respiratory system and in the whole organism systemically and in the lungs. The primary hypothesis was that the administration of IFN-β by inhalation could might be able to enhance innate immunity, thereby compensating for the known IFN-β relative deficiency, which was previously demonstrated ex vivo in the epithelium of patients with moderate–severe asthma. Along this line, IFN-β may have a convenient and favorable impact on virus-induced asthmatic exacerbations in difficult-to-treat patients, in whom the underlying disease is likely to be more severe with a higher risk of health impact [91]. The results of this trial suggest that IFN-β may represent a potential effective treatment for virus-induced deteriorations of asthma in difficult-to-treat people with asthma and justify further future clinical studies conducted in this high-risk population.

## 6. Conclusions

RV is a very common pathogen that causes upper and lower RTI in children and adults. In the last few decades, it was observed to be related to subsequent development of asthma and recurrent wheezing in childhood, as widely demonstrated by several epidemiological studies. Indeed, the immune response of the host against viral infection in the first months of life is primarily Th2-mediated, and this response may lead to bronchial hyper-responsiveness in predisposed patients. In this context, a rising number of studies report a predominant Th2 polarization of host response to RV-infection-associated bronchiolitis as compared to RSV or other etiological viral agents. The broad variety of RV genotypes and the poor cross-protection from previous exposure to heterologous infections represent a major obstacle to the development of specific antiviral agents and vaccines against RV. Ongoing research on RV’s structure, serotype-based peculiarities, and host factors that predispose to an asthmatic response may be useful to improve preventive and effective treatment strategies to limit the overall burden of RV disease, and the consequent risk of developing chronic respiratory morbidity in childhood.
